# Plants with Antimicrobial Activity Growing in Italy: A Pathogen-Driven Systematic Review for Green Veterinary Pharmacology Applications

**DOI:** 10.3390/antibiotics11070919

**Published:** 2022-07-08

**Authors:** Cristian Piras, Bruno Tilocca, Fabio Castagna, Paola Roncada, Domenico Britti, Ernesto Palma

**Affiliations:** 1 Department of Health Sciences, “Magna Græcia University” of Catanzaro, Campus Universitario “Salvatore Venuta” Viale Europa, 88100 Catanzaro, Italy; tilocca@unicz.it (B.T.); fabiocastagna@unicz.it (F.C.); roncada@unicz.it (P.R.); britti@unicz.it (D.B.); palma@unicz.it (E.P.); 2 Interdepartmental Center Veterinary Service for Human and Animal Health, “Magna Græcia University” of Catanzaro, CISVetSUA, Campus Universitario “Salvatore Venuta” Viale Europa, 88100 Catanzaro, Italy; 3 Department of Health Sciences, Institute of Research for Food Safety & Health (IRC-FISH), “Magna Græcia University” of Catanzaro, Campus Universitario “Salvatore Venuta” Viale Europa, 88100 Catanzaro, Italy; 4 Nutramed S.c.a.r.l., Complesso Ninì Barbieri, Roccelletta di Borgia, 88021 Catanzaro, Italy

**Keywords:** bioactive plants, antibacterial, antimicrobial, essential oil, Green Veterinary Pharmacology

## Abstract

Drug resistance threatening humans may be linked with antimicrobial and anthelmintic resistance in other species, especially among farm animals and, more in general, in the entire environment. From this perspective, Green Veterinary Pharmacology was proven successful for the control of parasites in small ruminants and for the control of other pests such as varroa in bee farming. As in anthelmintic resistance, antimicrobial resistance (AMR) represents one of the major challenges against the successful treatment of infectious diseases, and antimicrobials use in agriculture contributes to the spread of more AMR bacterial phenotypes, genes, and proteins. With this systematic review, we list Italian plants with documented antimicrobial activity against possible pathogenic microbes. Methods: The literature search included all the manuscripts published since 1990 in PubMed, Web of Science, and Scopus using the keywords (i) “antimicrobial, plants, Italy”; (ii) “antibacterial, plant, Italy”; (iii) “essential oil, antibacterial, Italy”; (iv) “essential oil, antimicrobial, Italy”; (v) “methanol extract, antibacterial, Italy”; (vi) “methanol extract, antimicrobial, Italy”. Results: In total, 105 manuscripts that documented the inhibitory effect of plants growing in Italy against bacteria were included. One hundred thirty-five plants were recorded as effective against Gram+ bacteria, and 88 against Gram−. This will provide a ready-to-use comprehensive tool to be further tested against the indicated list of pathogens and will suggest new alternative strategies against bacterial pathogens to be employed in Green Veterinary Pharmacology applications.

## 1. Introduction

Sustainable livestock management can achieve carbon neutrality thanks to greenhouse gas reabsorption by photosynthetic processes of plants used as feed [1]. However, carbon neutrality alone does not complete the circle of fully sustainable farming that still requires the use of chemically synthesized antimicrobial and anthelmintic drugs that may persist in the environment. To overcome this problem, Green Veterinary Pharmacology approaches promote the use of plants and natural products for pest control. These approaches have already been successfully applied for the control of parasites in small ruminants [2,3,4] and bee farming [5,6,7] providing a relevant alternative to conventionally used drugs whose efficacy is hampered by resistance phenomena [8,9].

After the current pandemics, the next challenge for humanity might be represented by antimicrobial resistance (AMR). The environment, including animals and animal products, is colonized by bacteria that are typical and specific to every different ecological niche. In these complex environments, natural and human-related ecological pressure promotes the selection and expression of genes related to AMR. AMR predates the clinical use of antibiotics, posing the question of whether AMR occurred earlier than human antibiotics production and spread [10,11]. For example, soil microorganisms are carriers of resistance genes to many classes of antibiotics independently from human-derived antimicrobial pressure. Naturally occurring AMR is related to the biological pressure of every ecological environment/niche that implicates the bacteria–bacteria competition or the bacteria–fungi competition. Therefore, bacteria–fungi co-existence may have been the driver for the initial production and synthesis of the early forms of beta-lactamases [10,11,12].

There are different possible intervention methods that can be used to avoid the spread and the threats related to antimicrobial resistance. At first, animals showing recurring resistance patterns in their microbiomes might be kept separated and culled. Another strategy may be represented by the intervention through Green Veterinary Pharmacology approaches by using crops and plants that produce molecules with antibacterial activity. Italian territory offers high biodiversity of endemic plants with the most diverse nutraceutical functions [13]. Part of this knowledge is embedded in the ancient traditions of rural territories and might be re-evaluated to scientifically confirm the eventual antimicrobial activity [14]. Another part is already recorded in the scientific literature and needs to be systematically resumed. Evaluating the effectiveness of these plants or their extracts on microbes that threaten the efficiency of animal production may represent a valid alternative to the common antimicrobial therapeutical procedures and may help reduce the development of further antibiotic resistance phenomena.

The aim of this review is to create a list (based on scientific knowledge) of the known autochthonous plants of Italian territory that could be used as alternative antimicrobial treatments in animal husbandry. This might represent the first step toward the use of natural products to contrast the growing phenomenon of antibiotic resistance in animal production.

## 2. Results

The literature search (see details in the methods section) yielded, in total, 577 entries that were filtered to 374 after duplicates and literature reviews were removed. Among those, 105 relevant articles were chosen through the Rayyan keywords filtering algorithm, manually validated, and included in the study. All the contributions (from 1990, publication date) involving virus, fungi, and other applications were discarded, and only experimental works involving Italian plant parts or extracts active against bacteria were included. The workflow was guided according to the PRISMA 2020 checklist as in Supplementary Material File S1.

**Figure 1:**
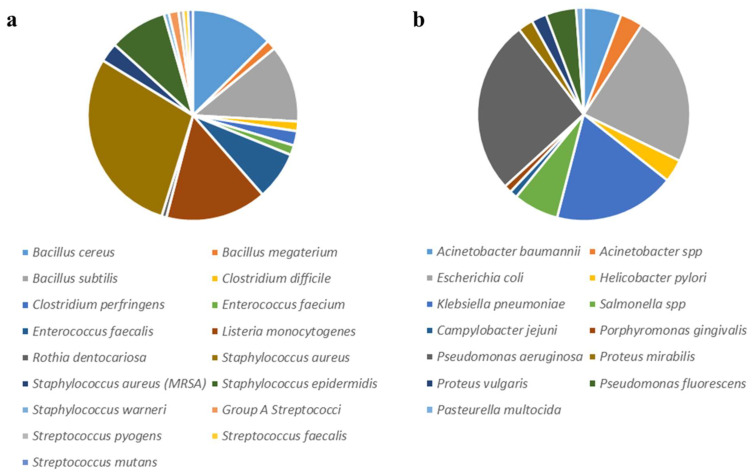
Pie chart showing the percentage of plants active against Gram+ (**a**) and Gram− (**b**).

Figure 1a shows the percentage of plants active against Gram+ bacterial, and Figure 1b shows the percentage of plants active against Gram− bacteria. Table 1 and Table 2 show the plants effective against each bacterial genera/species. Table 1 reviews the plants with documented antimicrobial activity on Gram+ bacteria, while Table 2 reviews the plants active towards Gram− bacteria. The results herein described were merged with the results presented in the systematic review published by Chassagne et al. in 2021 [15].

Among the plants effective against Gram+ bacteria, the major number of described species (39) was recorded for *S. aureus* (29%, Figure 1a), and, among these, four were specifically recorded as being effective against the MSSA strain. Twelve contributions (9%) were recorded for plants effective against *S. epidermidis*, 17 (13%) against *B. cereus,* and 21 (16%) against *Listeria monocytogenes*. Considering Gram−, 20 described plants were effective against *Escherichia coli* (23%), 23 against *P. aeruginosa* (26%), and 9 against *K. pneumoniae* (18%). Each percentage refers to the total number of plants effective against Gram+ or Gram− bacteria separately.

The most represented chemical classes included polyphenols (mainly tannins, 14 hits), terpenes (mainly limonene) with 6 relevant hits, flavonoids (24 hits), and alkaloids (6 hits).

Interestingly, plants were detected to be active against the growth of all ESKAPE pathogens, including *Enterobacter* spp., as reported in Table 2.

## 3. Discussion

Antimicrobial use in agriculture is partially responsible for the spread of AMR. Global deaths linked to AMR worldwide are estimated to increase up to 750,000 and are projected to reach as high as 10 million by the year 2050 [118].

Plant evolution developed strategies to ensure life adopting numerous effective defense mechanisms, such as the production of secondary metabolites to combat pests and pathogens [119,120], and these molecules could represent alternative solutions to go beyond the rise of antibiotic resistance.

Those secondary metabolites help plants fight stressor agents, interact with other organisms (herbivores, pathogens, neighboring plants, pollinators, and fruit dispersers), and are mainly part of three large chemical classes with relevant bioactivity as terpenes, phenols, and alkaloids. Among those, terpenoids represent one of the richest classes of molecules and include more than 50,000 known compounds. Many of these compounds have the function of defending the plant from possible bacterial pathogens.

With this review, we propose a pathogen-driven list of the plants that can grow in Italian territory with evidence of antibacterial activity. The list is ordered according to the bacterial pathogens to facilitate future studies for possible therapeutic approaches.

### 3.1. Plants Active against Gram+

*B. cereus* is of particular interest to public health because of food spoilage and toxin production [121]. Already in 2007, it was detected as a contaminant in cow feed, farm environment, and ultimately, in bulk milk [122]. As in Table 1, 17 plants or their extracts have been found to be effective against its growth, and most of those are very common and easy to find, such as *Laurus nobilis* [19], *Malus domestica* var. *Annurca* [21], *Allium sativum* L. [24], *Rosmarinus officinalis* L., *Lavandula angustifolia Miller* [25], *Sage (Salvia officinalis),* and *Thymus vulgaris* [28].

Clostridial diseases in farm animals may affect productivity and safety. Clostridium species are ubiquitous and populate the enteric flora of animals. They can be the cause of alimentary tract infections or be responsible for infections of tissues other than gastrointestinal [123,124]. Enterotoxemia type C, Enterotoxemia type D, and tetanus are common clostridial diseases affecting farm animals, and, in the Italian region, three plants (*Angelica archangelica* L. [41], *Satureja montana* [43], *Echinophora spinosa* [42] showed an inhibiting capability against *C. perfrigens* and *C. difficile*. The most abundant components of the EO extracted from *E. spinosa* plants growing in Italy are α-pinene (21.3%), δ-3-carene (16.5%), limonene (16.4%), and α-phellandrene (8.7%) [41].

Enterococci are bacteria that naturally colonize animals’ intestines. *E. faecalis* and *E. faecium* are part of the ESKAPE pathogens (*E. faecium*, *S. aureus*, *Klebsiella pneumoniae*, *A. baumannii*, *P. aeruginosa*, and *Enterobacter* species) and are relevant worldwide because they are responsible for an increasing number of nosocomial infections such as bacteremia and infectious endocarditis [125]. Pathogenic strains could carry that vanA gene cluster (Tn 1546) that encodes for vancomycin resistance [126] and, more in general, bacteria of the enterococci genus of animal origin are responsible for the flow of genes of antibiotic resistance from animals to humans [127]. As in Table 1, 12 different plants were recorded as being active against *E. faecalis* and *E. faecium.*

*S. aureus* infections in animals are mostly associated with mastitis in dairy-producing animals. Even if phylogenetic studies demonstrated that this pathogen tropism may be specific for animals and humans [128], antimicrobial pressure in livestock might lead to the selection of resistant strains and genes posing a risk for a jump of species [129]. According to our findings reported in Table 1, the Italian territory offers at least 39 plants with demonstrated activity against this pathogen. In addition, four plants (*Crinum angustum Steud.* [81], *Limonium avei (De Not.)* *Brullo* and *Erben* [31], *Cytinus hypocistis* [62], *Chiliadenus lopadusanus* [82]) were documented as active against the most pathogenic methicillin-resistant strain (MRSA).

### 3.2. Plants Active against Gram−

Another member of the ESKAPE pathogens is *Acinetobacter baumannii*. It represents a consistent cause of drug-resistant infections, and its resistance traits were found in many companion and food-producing animals such as dogs, cattle, sheep, and goats [130]. Three studies listed *Daucus carota* subsp. *Maximus* [95], *Lavandula × intermedia* [18] and *Cytinus* [62] as plants effective against the *Acinetobacter* genus, and another three studies documented *Chiliadenus lopadusanus* [82], *Cistus creticus (CC),* and *Cistus salviifolius (CS)* [93] as effective against *A. baumannii*. Among these, the *Daucus carota* plant is effective at a concentration ranging from 1.25 to 2.50 μL/mL, *Lavandula × intermedia* essential oil (pure) generated an inhibition zone of 47 mm, and *Cytinus* ethanolic and water extracts (0.5 mg/disc) showed an inhibition zone of around 10 mm.

Bacteria of the *Klebsiella (K.)* genus can be found in the environment, e.g., in soil and water [131]. *K. pneumoniae* is considered one of the most dangerous multi-drug resistant microorganisms [132] and, more than in the environment, it can be found in insects and in domestic and wild mammals [133].

Among farm animals, it can be the causative agent of pneumonia, epidemic metritis, cervicitis in mares, and septicemia in foals [134]. It can be the etiological agent of pneumonia and mastitis in bovines [135] and can cause losses in milk production, decreased milk quality, and higher mortality [136]. Its resistance gene products were found in bulk tank milk from a well-managed research facility at the University of Milan [137].

As in Table 2, nine different plants growing in Italian soil are effective against the growth of this dangerous pathogen. Among those, *Arbutus unedo* [105], *Myrtus comunis* [107] and *Pistacia lentiscus* [108] are certainly easy-to-find and recognized plants that could be used to improve animal welfare and help the fight against this pathogen.

Animals can be the reservoir of *P. aeruginosa* multi-drug resistant strains. Interestingly, the detected strains were found to be resistant to carbapenems even though that class of molecules was not employed for animal use [138]. Being able to control such infections may be useful to avoid the jump of those strains from animals to humans. Our work highlighted 23 different plants growing in Italian soil that could be used to counteract this pathogen. Among those plants, there are *Citrus* spp., which easily and commonly grow in the south of the Italian peninsula.

### 3.3. Pharmacodynamics of Plant Extracts

Phytocomplexes, by definition, represent a mixture of bioactive compounds that can act in synergy by targeting multiple receptors, facilitating the molecules towards their target, or slowing active molecule degradation [139,140]. 

The higher effectiveness of plant phytocomplexes rather than single molecules is demonstrated by the reduced activity after fractionation [139,140]. Moreover, it is now mainly accepted that there is a necessity for compounds that synergize with existing antibiotics to be used against drug-resistant bacteria [141,142,143].

Plant-based compound extracts can be helpful in the fight against antibiotic resistance. However, their eventual application should be carefully regulated and controlled to avoid the development of the growing resistance mechanisms to less specific biocides (antiseptics, disinfectants, and preservatives) [144].

## 4. Materials and Methods

All the literature entries, including the abstracts, were collected from PubMed, Web of Science, and Scopus. Every different database was queried with the following keywords: (i) “antimicrobial, plants, Italy”; (ii) “antibacterial, plant, Italy”; (iii) “essential oil, antibacterial, Italy”; (iv) “essential oil, antimicrobial, Italy”; (v) “methanol extract, antibacterial, Italy”; (vi) “methanol extract, antimicrobial, Italy”. The search parameters included all the documents published since 1990, and the keywords searches were restricted to the titles and the abstracts.

The results from the “PubMed” database were downloaded in the “PubMed” format. The results obtained using Scopus and Web of Science were downloaded in the RIS format.

All the output files were uploaded in the rayyan Systematics Reviews research tool (https://www.rayyan.ai/, accessed on 13 May 2022). The auto duplicates tool was used to remove duplicate entries, the reviews were excluded from the databases via the exclusion tool of rayyan, and obtained results were uploaded as new files to keep the review entries out. The remaining abstracts were manually evaluated for the inclusion decision. Only scientific products, including experimental work performed with plants or plant extracts documenting the bacterial growth inhibition potential, were included.

## 5. Conclusions

Plants and natural products have been widely used in the past against bacterial pathogens. Most of this knowledge is part of tradition and has been recently re-evaluated to gather complementary or alternative methods in place of antibiotic treatments. From this perspective, antimicrobial products of plant origin may represent a relevant solution to antibiotic resistance because of the simultaneous presence of diverse, active molecules such as secondary metabolites, terpenoids, alkaloids, and/or tannins. This heterogeneity of chemical compounds exploits its pharmacodynamic action via a multi-targeted approach [139] making the generation of resistance mechanisms more difficult.

This review collects, through a systematic approach, the knowledge about the plants growing in Italian territory active against bacterial pathogens. This tool may be useful to rapidly use the listed plants as possible candidates for further research purposes and for the treatment of bacterial infections of veterinary interest.

## Figures and Tables

**Table 1:** Plants active against Gram+ bacteria.

Bacterium (Gram+)	Number of Plant Species	Plant Name
*Bacillus cereus*	17	*Daucus carota subsp. maximus (Desf.)Ball* [16]; *Achillea moschata* [17]; *Lavandula* × *intermedia* [18]; *Laurus nobilis* [19]; *Dianthus rupicola* [20]; *Malus domestica* var. *Annurca* [21]; *Teucrium genus (Germander)* [22]; *Rapa Catozza Napoletana (Brassica rapa* L. var. *rapa DC.)* [23]; *Allium sativum* L. [24]; *Rosmarinus officinalis* L. and *Lavandula angustifolia Miller* [25]; *Guava (Psidium guajava)*, *Sage (Salvia officinalis)*, *Rhamnus (Ziziphusspina Christi)*, *Mulberry (Morusalba* L.*)*, and *Olive (Oleaeuropaea* L.*)* [26]; *Fuscoporia torulosa* [27]; *Thymus vulgaris* [28]; *Corylus avellana* [29]
*Bacillus megaterium*	2	*Origanum heracleoticum* and *O. majorana* [30]
*Bacillus subtilis*	16	*Limonium avei (De Not.) Brullo* and *Erben* [31]; *Schinus molle* (L.) [32]; *Thymus vulgaris* [28]; *Crocus sativus* L. [33]; *Glycyrrhiza glabra* [34]; *Dianthus rupicola* [20]; *Lavandula angustifolia* L. [35]; *Hypericum taxa (Guttiferae)* [36]; *Fuscoporia torulosa (Basidiomycetes)* [27]; *Fuscoporia torulosa* [27]; *Thymus vulgaris* [28]; *Bupleurum fontanesii* [37]; *Crithmum maritimum* [38]; *Ferulago campestris* [39]; *Origanum onites* and *Thymus capitatus* [40]
*Clostridium difficile*	2	*Angelica archangelica* L. [41]; *Echinophora spinosa (Apiaceae)* [42]
*Clostridium perfringens*	3	*Angelica archangelica* L. *(Apiaceae)* [41]; *Satureja montana* L. [43]; *Echinophora spinosa (Apiaceae)* [42]
*Enterococcus faecium*	2	*Cytinus hypocistis*, *Cytinus ruber* [44]
*Enterococcus faecalis*	10	*Schinus molle* (L.) [32]; *Achillea moschata* [17]; *Angelica archangelica* L. *(Apiaceae)* [41]; *Rapa Catozza Napoletana (Brassica rapa* L. var. *rapa DC.)* [23]; *Cinnamomum camphora* (L.) [45]; *Myrtus communis* [46]; *Arbutus unedo* L. [47]; *Echinophora spinosa (Apiaceae)* [42]; *Ferulago campestris* [39]; *Juniperus* spp. [48]
*Listeria monocytogenes*	21	*Ceratonia siliqua* L. [49]; *Daucus carota subsp.* *maximus (Desf.) Ball* [16]; *Limonium avei (De Not.) Brullo* and *Erben* [31]; *Centaurium erythraea* [50]; *Thymus vulgaris* L. [51]; *Cannabis sativa* [52]; *Lavandula* × *intermedia* and *Lavandula angustifolia* [53]; *Rapa Catozza Napoletana (Brassica rapa* L. var. *rapa DC.)* [23]; *Cinnamomum camphora* (L.) [45]; *Allium ampeloprasum* [54]; *Citrus taxa-Citrus medica*, *Citrus bergamia* [55]; *Conium maculatum*, *Apiaceae* [56]; *Allium sativum* L. [24]; *Schinus molle* (L.) [32]; *Cytinus* [44]; *Citrus medica* L. [57]; *Achillea moschata* [17]; Crithmum maritimum [38]; *Artemisia arborescens* [58]
*Rothia dentocariosa*	1	*Punica granatum* L. [59]
*Staphylococcus aureus*	39	*Cinnamomum* [60]; *Cinnamomum camphora* (L.) [45]; *Cistus monspeliensis* L. [61]; *Cistus salviifolius* L. [61]; *Cytinus hypocistis* (L.) L. [62]; *Limonium morisianum Arrigoni* [63]; *Myrtus communis* L. [64]; *Origanum vulgare* L. [65]; *Pistacia lentiscus* L. [66]; *Pistacia terebinthus* L. [67]; *Rosmarinus officinalis* L. [68]; *Salvia officinalis* L. [69]; *Thymus herba-barona Loise* L. [70]; *Thymus vulgaris* L. [71]; *Inula crithmoides* [72]; *Caralluma europaea* [73]; *Crocus sativus* [33]; *Helichrysum araxinum* [74]; *Schinus molle* (L.) [32]; *Cannabis sativa* [75]; *Centaurium erythraea* [50]; *Citrus medica* L., *Citrus bergamia*, and *Citrus medica* [55]; *Laurus nobilis* [19]; *Rubus ulmifolius* [76]; *Malus domestica* var. *Annurca* [21]; *Teucrium genus (Germander)* [22]; *Daucus carota subsp.* *maximus (Desf.)* [16]; *Cytinus* [44]; *T. vulgaris*, *S. montana* and *C. sativum* [77]; *Garlic (Allium sativum* L.*)* [24]; *Thymus vulgaris* L. [28]; *Rapa Catozza Napoletana (Brassica rapa* L. var. *rapa DC.)* [23]; *Calycotome villosa (Poiret)* [78]; *Juniperus* spp. [79]; *Hyssopus officinalis* [80]
*Staphylococcus aureus (MRSA)*	4	*Crinum angustum Steud.* [81]; *Limonium avei (De Not.)* *Brullo* and *Erben* [31]; *Cytinus hypocistis* [62]; *Chiliadenus lopadusanus* [82]
*Staphylococcus epidermidis*	12	*Arbutus unedo* L. [83]; *Cistus monspeliensis* L. [84]; *Cistus salviifolius* L. [85]; *Cytinus hypocistis* (L.) L. [62]; *Limonium avei (De Not.) Brullo* and *Erben* [31]; *Limonium morisianum Arrigoni* [63]; *Myrtus communis* L. [46]; *Pistacia lentiscus* L. [66]; *Cytinus.* [44]; *Thymus vulgaris* L. [28]; *Salvia adenophora* [86]; *Magydaris tomentosa* [87];
*Staphylococcus warneri*	1	*Daucus carota subsp. maximus (Desf.) Ball* [16]
*Group A Streptococci*	2	*Origanum* and *Thymus* [88]
*Streptococcus pyogens*	1	*Teucrium genus* [22]
*Streptococcus faecalis*	1	*Thymus vulgaris* L. [28]
*Streptococcus mutans*	1	*Punica granatum* L. [89,90,91]; *Achillea ligustica* [92]

**Table 2:** Plants active against Gram− bacteria.

Bacterium (Gram−)	Number of Plant Species	Plant Name
*Acinetobacter baumannii*	5	*Chiliadenus lopadusanus* [82]; *Cistus creticus (CC)* and *Cistus salviifolius (CS)* [93]; *Rumex crispus* L. and *Rumex sanguineus* [94]
*Acinetobacter* spp.	3	*Daucus carota subsp. maximus* [95]; *Lavandula × intermedia* [18]; *Cytinus hypocistis* [62]
*Enterobacter cloacae*	1	*Mentha* spp. [96]
*Escherichia coli*	20	*Daucus carota subsp. Maximus* [16]; *Cytinus hypocistis* [62]; *Matthiola incana (*L.*)* *R.Br. subsp. incana (Brassicaceae)* [97]; *Lavandula × intermedia* [18]; *Laurus nobilis* [19]; *Glycyrrhiza glabra* L. [34]; *Malus domestica* var. *Annurca* [21]; *Teucrium genus (Germander)* [22]; *Daucus carota subsp. maximus (Desf.)* [16]; *Isatis tinctoria* L. *(Brassicaceae)* [98]; *Garlic (Allium sativum* L.*)* [24]; *Thymus vulgaris* L. [28]; *Plectranthus barbatus* and *Plectranthus caninus* [99]; *Rapa Catozza Napoletana (Brassica rapa* L. var. *rapa DC.)* [23]; *Daphne gnidium* L. [100]; *Calycotome villosa* [78]; *Hyssopos officinalis* L. [80]; *Achillea ligustica* [101]; *Lupinus* spp. [102];
*Helicobacter pylori*	3	*Citrus* spp. [103]; *Cannabis sativa* L. [75]; *Apium nodiflorum (Apiaceae).* [104]
*Klebsiella pneumoniae*	16	*Arbutus unedo* [105]; *Cistus* spp. [93]; *Cytinus hypocistis* [62,106]; *Myrtus comunis* [107]; *Pistacia lentiscus* [108]; *Teucrium genus (Germander)* [22]; *Cytinus.* [44]; *Thymus vulgaris* L. [28]; *Pistacia terebinthus* [109]; *Rapa Catozza Napoletana (Brassica rapa* L. var. *rapa DC.)* [23]; *Crinum angustum* [110]; *Tinospora cordifolia* and *Alstonia scholaris* [111]; *Rhus coriaria* L. [112]; *Calycotome villosa* [78]; *Melaleuca alternifolia* [113]; *Mentha* spp. [96]
*Salmonella* spp.	6	*Origanum vulgare* [114]; *Lavandula* × *intermedia* and *Lavandula angustifolia* [53]; *Thymus vulgaris* L. [28]; *Rapa Catozza Napoletana (Brassica rapa* L. var. *rapa DC.)* [23]; *Mentha* spp. [96]
*Campylobacter jejuni*	1	*Artemisia annua* [115]
*Porphyromonas gingivalis*	1	*Pistacia lentiscus* L. [116]
*Pseudomonas aeruginosa*	23	*Cinnamomum camphora* [45]; *Allium ampeloprasum* var. *holmense Asch. et Graebn.* [54]; *Schinus molle (*L.*)* [32]; *Achillea moschata* [17]; *Citrus medica* L., *Citrus bergamia*, and *Citrus medica* [55]; *Centaurium erythraea* [50]; *Laurus nobilis* [19]; *Teucrium genus (Germander)* [22]; *Cytinus.* [44]; *Conium maculatum*, *Apiaceae* [56]; *Garlic (Allium sativum* L.*)* [24]; *Five Thymus vulgaris* L. [28]; *Rapa Catozza Napoletana (Brassica rapa* L. var. *rapa DC.)* [23]; *Lupinus* spp. [102]; *Calycotome villosa* [78]; *Juniperus* spp. [79]; *Allium ampeloprasum* [54]; *Allium sativum* [24]; *Melaleuca alternifolia* [113]; *Conium maculatum* [56]; *Achillea ligustica* [101]
*Proteus mirabilis*	2	*Rapa Catozza Napoletana (Brassica rapa* L. var. *rapa DC.)* [23]; *Hyssopos officinalis* L. [80]
*Proteus vulgaris*	2	*Thymus vulgaris* L. [28] *Rapa Catozza Napoletana (Brassica rapa* L. var. *rapa DC.)* [23]
*Pseudomonas fluorescens*	4	*Lavandula × intermedia* [18]; *Origanum heracleoticum* and *O. majorana* [30]; *Cannabis sativa* [117]
*Pasteurella multocida*	1	*Morus alba* [26]

## Data Availability

Not applicable.

## Supplementary Materials

The following are available online at https://www.mdpi.com/article/10.3390/antibiotics11070919/s1, File S1: PRISMA 2020 Checklist.
